# ﻿Morphological and phylogenetic analyses reveal two new species in *Conidiobolus* s.s. (Conidiobolaceae, Entomophthorales) from China

**DOI:** 10.3897/mycokeys.98.103603

**Published:** 2023-07-05

**Authors:** Yong Nie, Yue Cai, Heng Zhao, ZhengYu Zhou, ChangWei Zhao, XiaoYong Liu, Bo Huang

**Affiliations:** 1 Anhui Provincial Key Laboratory for Microbial Pest Control, Anhui Agricultural University, Hefei 230036, China Anhui Agricultural University Hefei China; 2 School of Civil Engineering and Architecture, Anhui University of Technology, Ma'anshan 243002, China Anhui University of Technology Ma'anshan China; 3 Department of Biological and Environmental Engineering, Hefei University, Hefei 230601, China Hefei University Hefei China; 4 Institute of Microbiology, School of Ecology and Nature Conservation, Beijing Forestry University, Beijing 100083, China Beijing Forestry University Beijing China; 5 College of Life Sciences, Shandong Normal University, Jinan 250014, China Shandong Normal University Jinan China

**Keywords:** Conidiobolaceae, microspore, morphology, new taxa, phylogeny

## Abstract

The genus *Conidiobolus* s.s. (Conidiobolaceae, Entomophthorales) has been delimited to accommodate members that produce microspores. Herein, morphological studies, combined with phylogenetic analysis based on the nuclear large subunit of rDNA (nucLSU), the mitochondrial small subunit of rDNA (mtSSU), and the elongation-factor-like gene (EFL) revealed two *Conidiobolus* s.s. species isolated from plant debris in China. *Conidioboluslongiconidiophorus***sp. nov.** is mainly characterised by its long primary conidiophores, while *Conidioboluspolysporus***sp. nov.** is diagnosed by 2–3 primary conidia arising from branched primary conidiophores. Phylogenetically, the former is grouped into a separate clade, while the latter is closely related to *C.incongruus*, but is morphologically distinguished by its larger primary conidia and branched conidiophores.

## ﻿Introduction

The genus *Conidiobolus* (Ancylistaceae, Entomophthorales) was divided into five genera, i.e. *Azygosporus* B. Huang & Y. Nie, *Capillidium* B. Huang & Y. Nie, *Conidiobolus* s.s. B. Huang & Y. Nie, *Microconidiobolus* B. Huang & Y. Nie, and *Neoconidiobolus* B. Huang & Y. Nie based on the molecular and morphological evidences (Nie et al. 2020a; Cai et al. 2021). Subsequently, three families were introduced to accommodate the above five genera based on molecular and genomic data. They were Capillidiaceae Y. Nie, Stajich & K.T. Hodge, Conidiobolaceae B. Huang, Stajich & K.T. Hodge, and Neoconidiobolaceae X.Y. Liu, Stajich & K.T. Hodge (Gryganskyi et al. 2022). The family Conidiobolaceae includes three genera, while Capillidiaceae and Neoconidiobolaceae include one genus each. The genus *Conidiobolus* s.s. belongs to the family Conidiobolaceae.

Unfortunately, the type species of *Conidiobolus*, *C.utriculosus* Brefeld, had been missing for a long time. Therefore, *C.coronatus* was proposed as the epitype of *Conidiobolus* s.s. due to its prominence as a pathogenic fungus, its global distribution, and its usage as a model organism for fungal evolution (Spatafora et al. 2016; Möckel et al. 2022). This genus includes 18 species and is the largest among related genera (Goffre et al. 2020; Nie et al. 2020b).

Notably, not all species of *Conidiobolus* s.s. produce microspores, making it difficult to recognize them without phylogenetic data. These include *C.dabieshanensis* (Nie et al. 2017), *C.iuxtagenitus* (Waters and Callaghan 1989), *C.margaritatus* (Huang et al. 2007), *C.taihushanensis* (Nie et al. 2020b) and *C.lichenicolus* (Srinivasan and Thirumalachar 1968). However, their other unique morphological characters and phylogeny could contribute to their suitable identification. Meanwhile, the key to *Conidiobolus* s.s. was provided to understand the relationship among this fungal group morphologically (Nie et al. 2020b).

This study aims to describe and illustrate two new species of *Conidiobolus* s.s. based on their morphology and phylogenetic analyses. This study also details the diagnostic characteristics for species that were not observed to produce microspores, and the diversity of *Conidiobolus* s.s. found in China.

## ﻿Materials and methods

### ﻿Isolation and morphology

Plant debris was collected from Guniujiang National Nature Reserve, Qimen County and Shitai County, and Huoli Mountain, Ma,anshan City, Anhui Province, and Yangtianshan National Forest Park, Shandong Province. The strains of *Conidiobolus* s.s. were isolated from plant debris following the previous described methods (Drechsler 1952; King 1976) and improved by Nie et al. 2012. Plant debris samples were placed into sterilized plastic bags. When they were transferred into the laboratory, the isolation was conducted immediately. Plant debris samples were cut into small pieces with scissors and tiled evenly on the Petri dishes cover, and incubated on inverted Petri dishes containing PDA media (potato 200 g, dextrose 20 g, agar 20 g, H_2_O 1 L) at 21 °C for 7 days.

The inverted Petri dishes were examined daily by a stereomicroscope (SMZ1500, Nikon Corporation, Japan). When a Conidiobolus-like fungus appeared, it was transferred to a new PDA plate to obtain a pure culture for morphological studies. The micro-morphological structure was observed using a light microscope (BX51, Olympus Corporation, Tokyo, Japan) and imaged using a microscope-camera system (DP25, Olympus Corporation, Tokyo, Japan). The morphological traits of the primary conidia and conidiophores, microconidia, resting spores etc. were described using the method by King (1976). All isolates were deposited at the Engineering Research Center of Biofilm Water Purification and Utilization Technology of Ministry of Education at Anhui University of Technology, Anhui Province, China (BWPU), and duplicated at the Research Center for Entomogenous Fungi at Anhui Agricultural University, Anhui Province, China (RCEF). A total of 14 ex-types of *Conidiobolus* s.l. were obtained from the American Type Culture Collection, Manassas, VA, USA (ATCC).

### ﻿DNA extraction, PCR amplification and sequencing

Genomic DNA was extracted from fresh fungal mycelia which were scraped from PDA, using a modified cetyltrimethylammonium bromide (CTAB) protocol as described in Watanabe et al. (2010). Three different loci were amplified using the following primer pairs: LR0R (5’-ACC CGC TGA ACT TAA GC-3’) / LR5 (5’-TCC TGA GGG AAA CTT CG-3’) for nucLSU (Vilgalys and Hester 1990), mtSSU1 (5’-GCW GCA GTG RGG AAT NTT GGR CAA T-3’) / mtSSU2R (5’-GTR GAC TAM TSR GGT ATC TAA TC-3’) for mtSSU (Zoller et al. 1999), and EF983 (5’-GCY CCY GGH CAY CGT GAY TTY AT-3’) / EF1aZ-1R (5’-ACA TCW CCG ACA CCC TTG ATC TTG -3’) for EFL (Nie et al. 2012).

Polymerase chain reaction (PCR) amplification reactions contained 1 μL dNTPs (200 μM), 1 μL MgCl_2_ (2.5 mM), 10 µL Phusion HF buffer (5x), 1 μL primers each (0.5 μM), 100 ng genomic DNA, and 0.5 μL Taq polymerase (0.04 Unit/L, Super Pfx DNA Polymerase, Cowinbioscience Co. Ltd., Shanghai, China). PCR amplificated program followed Nie et al. (2020b). Bi-directional sequencing was generated by Shanghai Genecore Biotechnologies Company (Shanghai, China). Sequences were processed with Geneious 9.0.2 (http://www.geneious.com, Kearse et al. 2012) to obtain consensus sequences. All sequences were deposited in GenBank (Table 1).

**Table 1.** The species used in phylogenetic analyses.

Species	Strains*	GenBank accession numbers		
		nucLSU	EFL	mtSSU
*Azygosporusmacropapillatus*	CGMCC 3.16068 (T)	MZ542006	MZ555650	MZ542279
*parvus*	ATCC 14634 (T)	KX752051	KY402207	MK301192
*Conidiobolusbifurcatus*	CGMCC 3.15889 (T)	MN061285	MN061482	MN061288
*C.brefeldianus*	ARSEF 452 (T)	EF392382	–	EF392495
*C.chlamydosporus*	ATCC 12242 (T)	JF816212	JF816234	MK301178
*C.coronatus*	NRRL 28638	AY546691	DQ275337	–
	RCEF 4518	JN131537	JN131543	–
*C.dabieshanensis*	CGMCC 3.15763 (T)	KY398125	KY402206	MK301180
*C.firmipilleus*	ARSEF 6384	JX242592	–	JX242632
*C.gonimodes*	ATCC 14445 (T)	JF816221	JF816226	MK301182
*C.humicolus*	ATCC 28849 (T)	JF816220	JF816231	MK301184
*C.incongruus*	NRRL 28636	AF113457	–	–
*C.iuxtagenitus*	ARSEF 6378 (T)	KC788410	–	–
	RCEF 4445	JX946695	JX946700	MK333391
*C.khandalensis*	ATCC 15162 (T)	KX686994	KY402204	MK301185
*C.lichenicolus*	ATCC 16200 (T)	JF816216	JF816232	MK301186
***C.longiconidiophorus* sp. nov.**	**RCEF 6563 (T)**	**OQ540746**	**OQ550509**	**OQ540744**
	**RCEF 6568 (T)**	**OR100884**	**OR113355**	**OR100881**
*C.macrosporus*	ATCC 16578 (T)	KY398124	KY402209	MK301188
*C.megalotocus*	ATCC 28854 (T)	MF616383	MF616385	MK301189
*C.mycophagus*	ATCC 16201 (T)	JX946694	JX946698	MK301190
*C.mycophilus*	ATCC 16199 (T)	KX686995	KY402205	MK301191
*C.polyspermus*	ATCC 14444 (T)	MF616382	MF616384	MK301193
***C.polysporus* sp. nov.**	**RCEF 4500**	**MG272478**	**MG272476**	**OR100882**
	**RCEF 7058 (T)**	**OQ540747**	**OQ550510**	**OQ540745**
*C.polytocus*	ATCC 12244 (T)	JF816213	JF816227	MK301194
*C.taihushanensis*	CGMCC 3.15900 (T)	MT250086	MT274290	MT250088
*C.variabilis*	CGMCC 3.15901 (T)	MT250085	MT274289	MT250087
*Microconidiobolusnodosus*	ATCC 16577 (T)	JF816217	JF816235	MK333388
*M.paulus*	ARSEF 450 (T)	KC788409	–	–
*M.terrestris*	ATCC 16198 (T)	KX752050	KY402208	MK301199

*ARSEF, ARS Entomopathogenic Fungus Collection (Ithaca, U.S.A.). ATCC, American Type Culture Collection (Manassas, U.S.A). CGMCC, China General Microbiological Culture Collection Center (Beijing, China). NRRL, ARS Culture Collection (Peoria, U.S.A). RCEF, Research Center for Entomogenous Fungi (Hefei, China). T = ex-type. The new species reported in this study are indicated in bold.

### ﻿Phylogenetic analyses

According to our previous studies (Nie et al. 2020a, b), the sequences of three loci (nucLSU, mtSSU, and EFL) of *Conidiobolus* s.s. species were retrieved from GenBank. Two *Azygosporus* and two *Microconidiobolus* species were chosen as out groups. Newly generated sequences from the three strains were aligned with all reference sequences by MAFFT program (Katoh and Standley 2013) and manually corrected with BioEdit (Hall 1999). The final alignments of three loci were concatenated using SequenceMatrix (Vaidya et al. 2011). The output sequence matrix was deposited in TreeBase (https://treebase.org) with the submission ID 30475. Maximum Likelihood (ML) and Bayesian Inference (BI) phylogenetic analyses were conducted. The best-fit substitution model of each partition was evaluated by MrModeltest 2.3 (Nylander 2004). The ML phylogenetic analysis was statistically tested in RAxML 8.1.17 with 1000 bootstrap replicates (Stamatakis 2014). The BI phylogenetic analyses included four MCMC chains and ran for 0.50 million generations until the average standard deviation of split frequencies was below 0.01. The trees were saved once every 100 generations. The burn-in fraction was set to 0.25 and posterior probabilities (PP) were determined from the remaining trees. Phylogenetic trees were checked with FigTree 1.4 (Rambaut 2012) and modified with Adobe Illustrator CS6.0 and Adobe Photoshop CS3.0.

## ﻿Results

### ﻿Phylogenetic analyses

**Figure 1.**
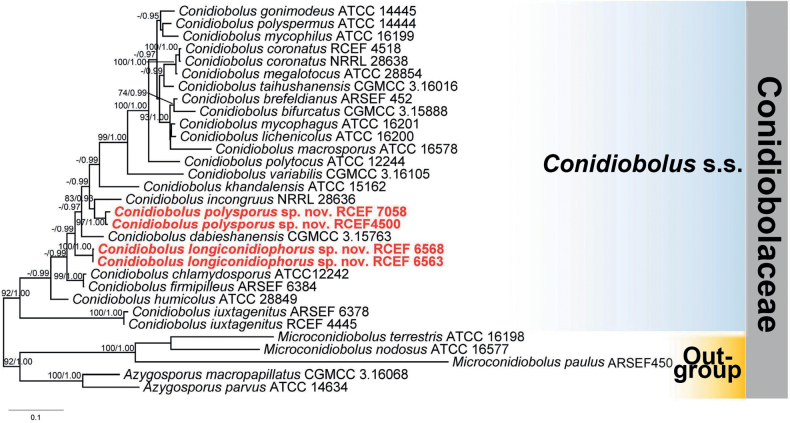
The phylogenetic tree of *Conidiobolus* s.s. constructed based on combined nucLSU, EFL and mtSSU sequences. *Azygosporus* and *Microconidiobolus* species were used as outgroups. New species are shown in red. Maximum Likelihood bootstrap values (≥70%) / Bayesian posterior probabilities (≥0.95) of clades are provided alongside the branches. The scale bar at the bottom left indicates substitutions per site.

The concatenated alignment utilized in this study comprised 1899 characters of nucLSU (1–984), EFL (985–1485), and mtSSU (1486–1899), out of which 986 characters are constant, 289 characters were found to be parsimony-uninformative and 624 characters were parsimony-informative. The best substitution model GTR+I+G was chosen for all the partitions during the ML and BI phylogenetic analyses. The final average standard deviation of the split frequencies was 0.00841, and the BI tree topology was found to be similar to that of ML. Therefore, the best scoring RAxML tree was used to represent the phylogenetic relationships among the studied taxa, with a final likelihood value of -13552.35 (Fig. 1). The phylogeny demonstrated that the three strains RCEF 4500 / 6563 / 6568 / 7058 in the present study were grouped with were members of the genus *Conidiobolus* s.s, revealing that the three strains belong to the family Conidiobolaceae, the genus *Conidiobolus* s.s. Furthermore, the strain RCEF 6563 and RCEF 6568 were grouped in an independent clade with a sound support (-/ 0.99), while the strains of RCEF 4500 and RCEF 7058 were claded with *C.incongruus* with a higher support (83 / 0.98).

### ﻿Taxonomy

#### ﻿ Conidiobolus longiconidiophorus

Taxon classificationFungiEntomophthoralesConidiobolaceae

B. Huang & Y. Nie sp. nov.

F8500F1D-73FF-5713-AEB0-B5AEC73E8448

MycoBank No: MB84768

Fig. 2

##### Etymology.

*Longiconidiophorus* (Lat.), referring to the long size of its conidiophores.

##### Known distribution.

Anhui Province, China.

##### Typification.

China, Anhui Province, Huangshan City, Qimen County, Guniujiang National Nature Reserve, 30°2′84′′N, 117°53′31′′E, from plant debris, 12 Dec. 2019, *Y. Nie and W. Wang*, holotype BWPU 191212. Ex-type culture RCEF 6563. GenBank: nucLSU = OQ540746; EFL = OQ550509; mtSSU = OQ540744.

##### Additional specimens examined.

China, Anhui Province, Chizhou City, Shitai County, Guniujiang National Nature Reserve, 30°10’66"N, 117°50’4"E, from plant debris, 15 Dec. 2019, *Y. Nie and W. Wang*, culture RCEF 6568. GenBank: nucLSU = OR100884; EFL = OR113355; mtSSU = OR100881.

##### Description.

Colonies on PDA at 21 °C after 3 d white, reaching ca 15 mm in diameter. Aerial hyphae flourishing after 6 d. Mycelia white, 5–10 μm wide, often unbranched at the edge of colony. Primary conidiophores often evolving from aerial hyphae, long, 150–340 × 6–9 μm, unbranched and producing a single primary conidium, without widening upward near the tip. Primary conidia forcibly discharged, globose, obovoid to ellipsoidal, 31–49 × 24–42 μm, papilla tapering and pointed, 7–13 μm wide, 3–7 μm long. Secondary conidiophores short or long, arising from primary conidia, bearing a single similar replicative conidium to primary conidia. Microspores not observed on the 2% water agar, but the structure similar to sterigmatas bearing microspores observed. Resting spores absent after 1 month.

##### Notes.

*Conidioboluslongiconidiophorus* forms a distinct phylogenetic clade from other *Conidiobolus* s.s. species. Morphologically, its primary condia are similar in size to those in *C.coronatus* (Cost.) Batko (14.5–38.5 × 17–48.5 μm), *C.dabieshanensis* Y. Nie & B. Huang (29–38 × 32.5–45), *C.macrosporus* Srin. & Thirum. (38–45 × 48–54 μm), *C.megalotocus* Srin. & Thirum. (30–50 μm), and C.utriculosus Brefeld (25–35 × 37.5–51 μm). However, it can be distinguished from *C.coronatus* and *C.macrosporus* by its longer primary conidiophores and the absence of resting spores (Batko 1964; Srinivasan and Thirumalachar 1967). Additionally, it is differentiated from *C.dabieshanensis* and *C.utriculosus* by its obovoid and ellipsoidal primary condia, as well as the absence of resting spores (Brefeld 1884; Nie et al. 2017). While it is closely related to *C.megalotocus*, it can be differentiated by the shape of its primary condia (Srinivasan and Thirumalachar 1962). Furthermore, in the phylogenetic tree (Fig. 1), *C.longiconidiophorus* is found to be distantly related to *C.megalotocus*.

**Figure 2.**
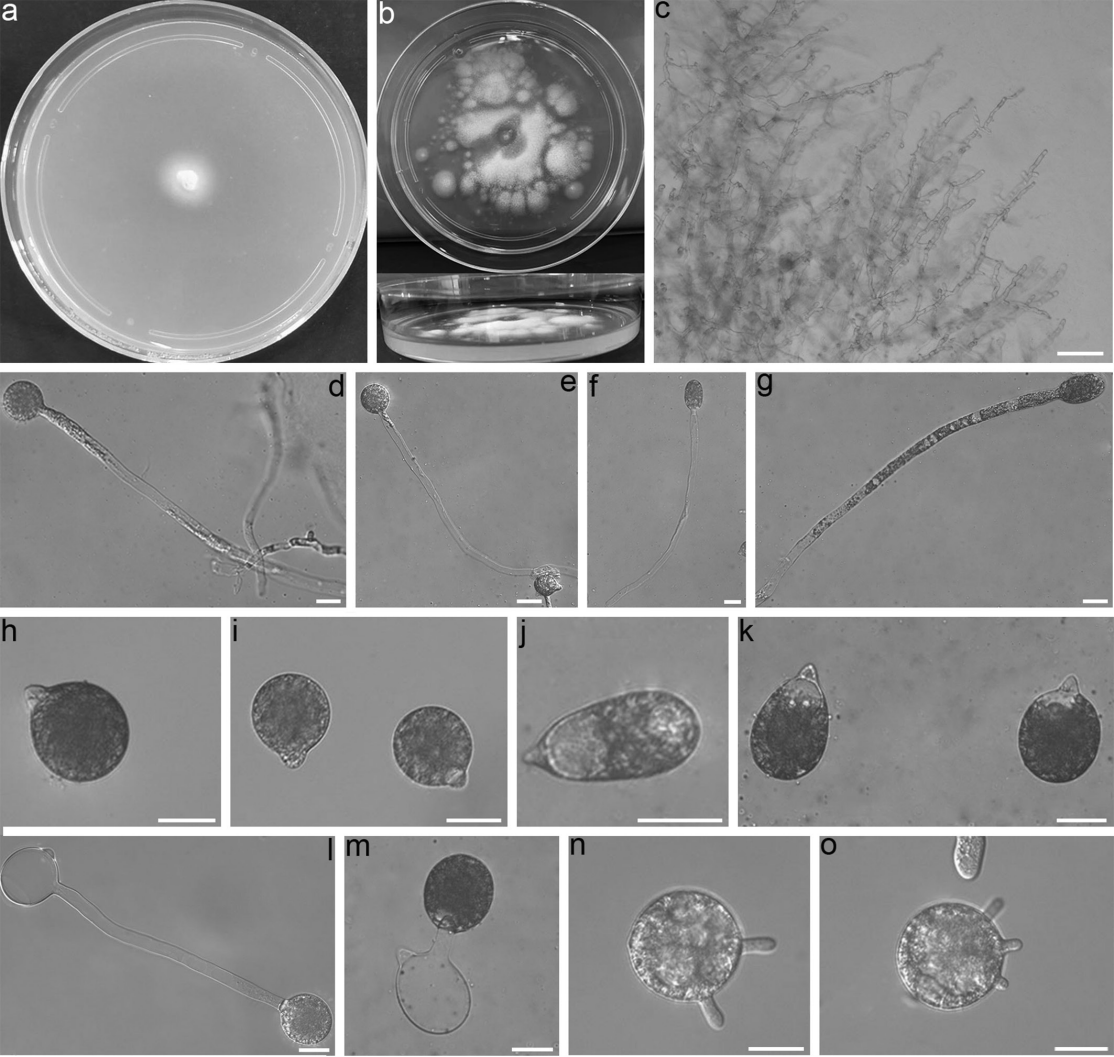
*Conidioboluslongiconidiophorus*RCEF 6563 **a** colony on PDA after 3 d at 21 °C **b** colony on PDA after 6 d at 21 °C **c** mycelia unbranched at the edge of the colony **d–g** primary conidiophores bearing primary conidia **h, i** globose primary conidia **j, k** obovoid to ellipsoidal primary conidia **l, m** primary conidia bearing a single secondary conidium **n, o** structure similar to sterigmatas arsing from conidia. Scale bars: 100 μm (**c**); 20 μm (**d–o**).

#### ﻿ Conidiobolus polysporus

Taxon classificationFungiEntomophthoralesConidiobolaceae

B. Huang & Y. Nie sp. nov.

63E76A6D-C6F1-5A09-A6E9-65ADE6263480

MycoBank No: MB84769

Fig. 3

##### Etymology.

*Polysporus* (Lat.), referring to several primary conidia arising from branched primary conidiophores.

##### Known distribution.

Anhui and Shandong Provinces, China.

##### Typification.

China, Anhui Province, Ma,anshan City, Huoli Mountain, 31°67′5′′N, 118°55′37′′E, from plant debris, 3 Nov. 2021, *Z.Y. Zhou and C.W Zhao*, holotype BWPU 211103. Ex-type culture RCEF 7058. GenBank: nucLSU = OQ540747; EFL = OQ550510; mtSSU = OQ540745.

##### Additional specimens examined.

China, Shandong Province, Qingzhou City, Yangtianshan National Forest Park, 36°46’31"N, 118°32’56"E, from plant debris, 18 Mar 2009, *C.F. Wang*, culture RCEF 4500. GenBank: nucLSU = MG272478; EFL = MG272476; mtSSU = OR100881.

##### Description.

Colonies on PDA at 21 °C after 3 d white, reaching ca 20–23 mm in diameter. Mycelia colorless, rarely branched at the edge of colony, 8.8–13 μm wide, vegetative hyphae filamentous, frequently appearing pronouncedly vacuolated, 15–22 μm wide. Primary conidiophores often unbranched, producing a single primary conidium, without widening upward near the tip, but in some instances bifurcate thus bearing two primary conidia, or forming three conidiophores at the tip thus bearing three primary conidia, 68–270 × 11–19 μm. Primary conidia forcibly discharged, mostly globose, 42–55 × 33–45 μm, Papilla 7.5–14 μm wide, 4–12 μm long. Sometimes obovoid, up to 65 μm long. Secondary conidia arising from primary conidia, similar and smaller to the primary conidia. Microconidia rarely observed on the 2% water agar, globose to elongate ellipsoidal, 7.5–8.8×7.5–12.5 μm. Zygospores formed between adjacent segments after 15 days, smooth, mostly globose, less often ellipsoidal, 17.5–37 μm in diameter, with a 1–3 μm thick wall.

##### Notes.

*Conidioboluspolysporus* is characterized by several primary conidia (2–3) arising from conidiophores, which are similar to those in *C.polytocus* Drechsler and *C.taihushanensis* B. Huang & Y. Nie. However, *C.polysporus* has larger primary conidia (42–55 × 33–45 μm in *C.polysporus* vs. 14–29 × 12–25 μm in *C.polytocus*), and forms zygospores while resting spores are absent in *C.polytocus* (Drechsler 1955). In addition, *C.polysporus* differs from *C.taihushanensis* due to its larger primary conidia (42–55 × 33–45 μm in *C.polysporus* vs. 27–42 × 19–32 μm in *C.taihushanensis*) and smaller zygospores (17.5–37 μm in *C.polysporus* vs. 34–48 × 23–40 μm in *C.taihushanensis*) (Nie et al. 2020b). Moreover, it is distantly related to *C.polytocus* and *C.taihushanensis* in the phylogenetic tree (Fig. 1). Although *C.polysporus* is grouped with *C.incongruus*, it can be distinguished by its larger primary conidia (42–55 × 33–45 μm in *C.polysporus* vs. 18–42 × 13–37 μm in *C.incongruus*) and branched conidiophore (Drechsler 1960).

**Figure 3.**
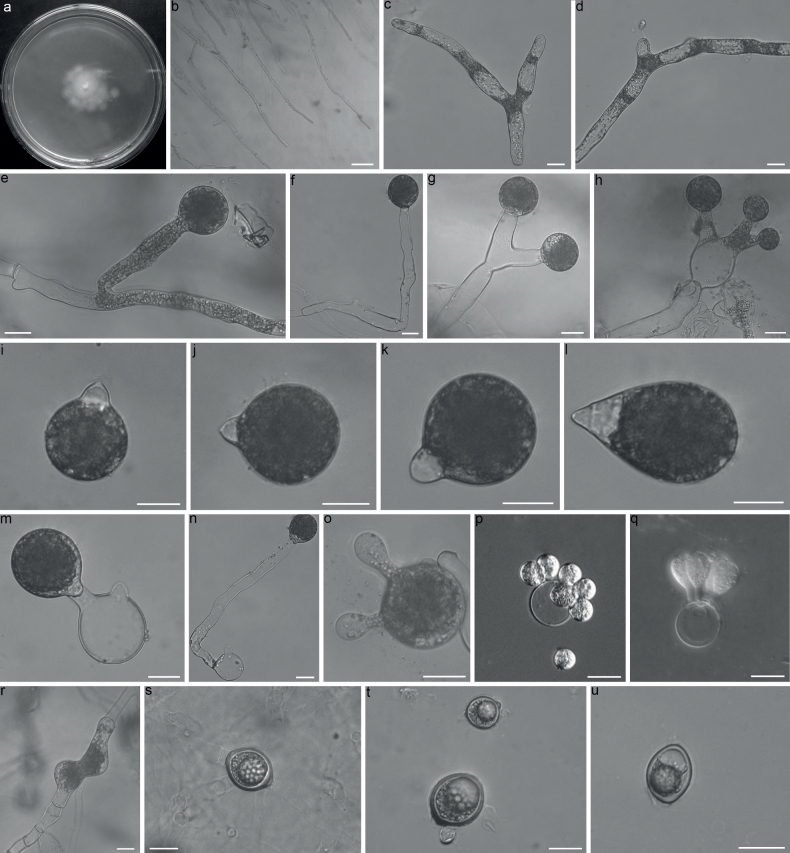
*Conidioboluspolysporus*RCEF 7058 **a** colony on PDA after 3 d at 21 °C **b** mycelia unbranched at the edge of the colony **c, d** hyphae appearing pronouncedly vacuolated **e, f** unbranched primary conidiophores **g, h** branched primary conidiophpores **i–k** globose primary conidia **l** obovoid primary conidia **m, n** primary conidia bearing a single secondary conidium **o–q** microconidia arising from a conidium **r, s** zygospores formed between adjacent segments of the same hypha **t, u** zygospores. Scale bars: 100 μm (**b**); 20 μm (**c–u**).

## ﻿Discussion

Although the family Conidiobolaceae was originally proposed to include three genera, *Azygosporus*, *Conidiobolus* s.s., and *Microconidiobolus*, recent phylogenetic analyses by Gryganskyi et al. (2022) revealed that the genus *Microconidiobolus* should be placed in a separate clade (Gryganskyi et al. 2022). In addition, we found that this fungal group produces smaller primary conidia, mostly less than 20 μm in size, without microspores, in comparison to most members of *Azygosporus* and *Conidiobolus* s.s. (Nie et al. 2020a). Therefore, it may be appropriate to recognize *Microconidiobolus* as a distinct family rather than a genus in the family Conidiobolaceae. However, additional evidence, including unique morphological characteristics, phylogenetic analyses with more taxa, and more genome data, is necessary to confirm this hypothesis. *C.longiconidiophorus* produces long primary conidiophores (over 300 μm) because most of them develop from aerial hyphae. We noticed that *C.dabieshanensis* (Nie et al. 2017) also produces such long primary conidiophores (up to 287 μm), and they are closely grouped together in the phylogenetic tree (Fig. 1). Coincidentally, these two species were not observed to produce microspores. Nevertheless, we made several attempts, such as culturing at low or high temperatures, on different culture media, and even exposing them to ultraviolet radiation to induce microspore formation. However, we were still unable to observe microspores, and we hypothesized that microspores of these species may only arise under the natural environment. This phenomenon was also observed in four other *Conidiobolus* s.s. species and may require further investigation.

*Conidioboluspolysporus* is known to produce 2–3 primary conidia arising from branched primary conidiophores. Similar branched primary conidiophores have also been observed in *C.gonimodes* (Drechsler 1961), *C.margaritatus* (Huang et al. 2007), *C.polytocus* (Drechsler 1955) and *C.taihushanensis* (Nie et al. 2020b). However, the number of primary conidia borne on these branched primary conidiophores varies: *C.gonimodes* and *C.margaritatus* produce 2 primary conidia, *C.polytocus* produces 2–4 primary conidia, and *C.taihushanensis* produces 2–6 primary conidia. Notably, the two primary conidia of *C.gonimodes* arise directly from the top of branched primary conidiophores without short handles (Drechsler 1961). Additionally, *C.polysporus* produces primary conidia that are larger than those produced by the other four *Conidiobolus* s.s. species mentioned above.

Interestingly, we found that *C.iuxtagenitus* was located at the bottom of the phylogenetic tree and was distinct from other *Conidobolus* s.s. members. *C.iuxtagenitus* is characterized by an absence of microspore and its zygospores formed by a short beak near a lateral conjugation (Waters and Callaghan 1989). Therefore, it is possible that *C.iuxtagenitus* represents another potential new lineage.

In this study, we introduce two new species of *Conidiobolus* s.s. species, namely *C.longiconidiophorus* and *C.polysporus*, based on morphological and phylogenetic evidence. These findings expand the number of known *Conidiobolus* s.s. species to 20.

## Supplementary Material

XML Treatment for Conidiobolus longiconidiophorus

XML Treatment for Conidiobolus polysporus

## Appendix

This study was supported by the National Natural Science Foundation of China (Nos. 31900008, 30770008 and 31970009). We also thank the editor and anonymous reviewer for their valuable comments.

## ﻿Additional information

### Author ORCIDs

Yong Nie https://orcid.org/0000-0001-8964-1661

Yue Cai https://orcid.org/0000-0002-4970-9673

Heng Zhao https://orcid.org/0000-0003-2938-5613

ZhengYu Zhou https://orcid.org/0000-0001-9312-1626

ChangWei Zhao https://orcid.org/0009-0007-7573-346X

XiaoYong Liu https://orcid.org/0000-0002-8808-010X

Bo Huang https://orcid.org/0000-0001-6032-7396

### Data availability

All of the data that support the findings of this study are available in the main text.
